# Ethical Design of a Robot Platform for Disabled Employees: Some Practical Methodological Considerations

**DOI:** 10.3389/frobt.2021.643160

**Published:** 2021-08-19

**Authors:** Tommaso Colombino, Danilo Gallo, Shreepriya Shreepriya, Yesook Im, Seijin Cha

**Affiliations:** ^1^ Naver Labs Europe, Grenoble, France; ^2^ Naver Labs, Seoul, South Korea

**Keywords:** robotic platforms, workplace studies, ethnography, user experience design, assistive technology

## Abstract

This paper explains the process of developing a scenario involving the use of a robotic platform to enhance the work experience of disabled employees. We outline the challenges involved in revealing the potential unintended consequences of introducing elements of Artificial Intelligence, automation, and robotics into a socially and ethically complex and potentially fragile scenario, and the practical challenges involved in giving a voice to vulnerable users throughout the design process. While an ideal case scenario would involve the disabled employees as much as possible directly in the design process, this can, realistically, be a challenge. In this paper, we detail a methodological and analytic approach that is centered around ethnography and design fictions. It is designed to provide a deeper understanding of all the stakeholders involved in the scenario while encouraging ethical reflection. Based on our findings, we argue that, while it is relatively easy to adopt an *a priori* ethical stance through notions such as inclusivity and accessibility, there are risks involved in making such *a priori* prescriptions with respect to the perspectives of different stakeholders in an applied research project. More specifically, we highlight the importance of understanding the broad organizational and bureaucratic characteristics of a business or workplace when devising HRI scenarios and tasks, and of considering elements such as business models, operating philosophy, and organizational hierarchies in the design process.

## Introduction

Extensive research has been conducted on designing robots that successfully support people with disabilities. A great deal of research is dedicated to the use of robots as therapeutic aids in controlled experimental or clinical environments. These studies leverage the fact that robots lend themselves well to repetitive tasks and can be used in training scenarios to teach specific skills (Javed et al., 2018).

HRI scenarios used to test therapy protocols can also be used to investigate and test cognitive, social and intellectual abilities and characteristics of specific disabilities (Hautop Lund, 2009). As in therapeutic scenarios, researchers leverage the suitability of robots for repetitive tasks, and their potentially non-threatening nature. Anthropomorphic robots or robots with facial features are used as proxies for humans to practice emotion recognition skills, under the assumption that they are “easier” to interact with and may boost engagement.

Outside of clinical scenarios, HRI offers the potential for robots to be used in the care and assistance of people with disabilities. These are people with physical disabilities and the elderly as well as people with developmental disabilities (DDs). Moreover, it is necessary to include the needs of many actors (Yamazaki et al., 2016), including those of caregivers. Robots may be used to assist caregivers or directly replace them, which may be desirable because the elderly and disabled may value their independence (Shiomi et al., 2014).

While experimental and clinical scenarios are of interest to the HCI community, we consider the workplace an equally important setting. Work integration is one of the biggest challenges faced by people with DDs (Dibia et al., 2015). While many countries have legislation mandating companies to employ a quota of disabled workers, the categories are broad and people with DDs may find it difficult to find gainful and interesting employment opportunities (Gaudion, 2016; The National Autistic Society, 2016). There is a lack of support, both when finding employment and during employment itself. It is also sometimes difficult for prospective employers to evaluate the true skill level and potential of employees with disabilities and to provide an environment that is adapted to their needs.

Previous research has studied the use of robotic support to enable employees with DDs to perform specific tasks (Baxter et al., 2018; Kidal et al., 2018; Stöhr et al., 2018; Kidal et al., 2019). However, to the best of our knowledge there is less focus on understanding the impact of organizational roles and characteristics on the definition of the robotic support. We believe that this is a critical aspect to consider in the design process in order to propose solutions that can realistically be implemented.

The research at the heart of this paper was conducted in collaboration with a Korean business that employs people with cognitive and developmental disabilities across a variety of business-to-business service operations. The goal of the study was to contribute to the development of scenarios involving the use of a robotic platform to enhance the work experience of the disabled employees. We used design fictions to elicit future scenarios and better understand the impact of using robotic technology from different stakeholders’ perspectives. While we were not looking to promote overly advanced visions of what robots might achieve, these futuristic visions helped us understand the expectations of our stakeholders. The aim of our project was to manage these expectations and work within the current state of the art to deliver a design proposal that could be realistically integrated into a workplace within 1 or 2 years.

We were quite conscious of ethical concerns and risks related to forcing technological innovation onto a potentially vulnerable population (disabled employees). Furthermore, as representatives of a research organization involved in AI and the design of robotic platforms and services, we knew the project would be characterized by a strong technology push. In particular, we were conscious of the fact that the push would involve not only the desire to put our own specific technology at the center of the “solution” to whatever design challenge we might identify, but also to view the introduction of a robotic platform in a work environment as an inherently positive intervention. We also knew that we would be managing more than one organizational configuration of disability: our own as HCI researchers, that of the organization that employs the disabled workers, that of the customers on the receiving end of the provided service, and that of the employees themselves, with the latter having potentially the weakest direct representation in the design process (Mankoff et al., 2010). While we did have some access to the employees through observational work, the language barrier and the reluctance of their managers to engage them directly in a participatory design experiment meant that we would have to make design decisions on their behalf. It should be observed that the managers at the outset of the project had no reason to give us unfettered access to employees whose emotional and professional wellbeing they are responsible for, and that they would consider representing their interests in the research activity as their professional responsibility.

In this paper, we outline how we approached the challenge of bringing together the perspectives and concerns of a variety of different stakeholders around future design scenarios, and how throughout that process we tried to uncover and address the risk of the unintended consequences of introducing elements of AI, automation and robotics into a socially and ethically complex and potentially fragile scenario. We do not presume in doing so to bring any kind of privileged understanding of ethical principles that would apply in general to the deployment of autonomous agents in the wild. Standards for ethical principles in AI are by and large agreed upon and documented (IEEE, 2019) which we do not focus on improving here. What we do want to focus on are some of the practicalities of ethical design in collaborative projects. Principles like the ones defined by the IEEE Global Initiative are sound, but are also created within a specific community of practice which many of the stakeholders in our project (and arguably in most applied research projects) are not a part of. When it comes to a general principle like well-being, and particularly in this case the well-being of disabled employees, we question who has the moral and practical authority to decide which actual features, what degree of automation and which changes to an existing workflow will best embody it?

In this paper, we attempt to document the process we underwent in order to try to bring ethical considerations within the practice of a collaborative, multi-stakeholder project. We discuss specific methodological and analytic approaches that we used in two phases of the project. In the first instance, we sought to gain a broad understanding of the organizational principles of our commercial partner. This includes what they consider from their own point of view to be their mission, their responsibilities towards their employees and their well-being, and how they practically set out to accomplish them. In a second phase of the project, we engaged the stakeholders in a speculative design exercise with a goal of bringing their respective ethical considerations and points of view out in open discussion, and attempt to define a broad scenario which could be agreed upon by all. The specific methodologies we used (ethnography and futuristic auto-biographies) are less important than the overall intent, even though these particular methods were well-suited to our purpose and we will therefore describe them in some detail.

The scenario that was ultimately agreed upon and that is described here has not, at the time of writing, been implemented, so we do not have evidence to present of an ultimately successful outcome. One of the reasons we document the process of trying to practically incorporate ethical principles in the preliminary study and participatory design phases of the project is that the relationship between researchers and the technologies they develop often ends once the technology is released in the wild (Colombino et al., 2019). A longitudinal study of the impact of a new technology in a workplace could prove our ethical considerations to have been right or wrong, and provide valuable insights for future projects. But once ownership of a technology is transferred it may also be impossible to intervene further, which makes it essential that we try to anticipate problems and understand how a technology will be appropriated before it is implemented.

## Related Work

In this section, we first discuss previous studies on robots, their roles, and perceptions in the context of workplace collaboration. We also discuss previous research on the use of robots around people with cognitive disabilities. Second, we discuss research on values, and design activities used in HCI and HRI.

### Robots in Workplaces

Robots can be found in many workplaces: from order-picking robots in warehouses, delivery robots on university campuses, to bomb disposal robots working alongside teams of soldiers (Royakkers and van Est, 2015). Robots have been used to guide visitors in public places such as museums and airports (Burgard et al., 1999; Fong et al., 2003; Kuno et al., 2007; Kuzuoka et al., 2008). Robots in workplaces can be divided into three broad categories of pre-programmed (e.g., industrial), tele-operated (e.g., drones) and autonomous robots. Autonomous robots are able to sense their environment and act with purpose. Examples include delivery robots in hospitals that distribute and register patients’ medicines (Smids et al., 2019).

In previous research on robots being introduced among people in workplaces (Mutlu and Forlizzi, 2008; Dietsch, 2010; Smids et al., 2019), it was found that robots may affect social settings and be experienced as displaying social behavior simply by being and acting among people. How a robot is perceived affects its adoption. Research has found that people unconsciously give the robot human characteristics (Forlizzi and DiSalvo, 2006) or even pet characteristics (Sirkin et al., 2016). The role and perception of autonomous robots have been studied extensively in the context of hospitals. In studies of hospital delivery robots, it is found that a range of factors influence people’s perception. The same robots are perceived differently in different hospital units such as postpartum and medical units (Mutlu and Forlizzi, 2008). In one study (Ljungblad et al., 2012), people described the robot as an alien worker or work partner. In other studies, some employees anthropomorphized the robot, whereas others regarded the robot as a machine (Morkes et al., 1999; Siino and Hinds, 2005).

Robots also help to reduce costs and alleviate the complexity of workflows, for example by reducing physical distance, through the deployment of nursing assistant robots (Chen and Kemp, 2010), and courier robots (Evans et al., 1992; Mutlu and Forlizzi, 2008). Researchers conclude that organizational factors such as workflow, political, social, emotional and environment perspectives are related to perceptions (Crawford et al., 1998). Overall, studies suggest that robots in work environments should be designed to respect organizational constraints, facilitate collaboration and willingness to work, while integrating social aspects.

In the context of people with cognitive disabilities, a great deal of research in HRI is dedicated to the use of robots as therapeutic aids in controlled experimental or clinical environments (Javed et al., 2018; Hautop Lund, 2009). Researchers have studied the use of robotic support to enable employees with DDs to perform specific tasks (Baxter et al., 2018; Kidal et al., 2018; Stöhr et al., 2018; Kidal et al., 2019) and propose task-sharing approaches with collaborative robots in the context of an industrial assembly line job in production facilities. Such research shows that often human workers maintain control over the flow of actions and decision making in the face of unexpected situations, while robots execute repetitive tasks. This sharing of tasks is difficult when workers have cognitive disabilities, so this research inverts the traditional role of task-sharing between humans and robots and proposes the concept of the robot as supervisor. However, this research does not take into account the views of stakeholders.

While existing work studied robots in the areas of health and care for people with DDs (Shukla et al., 2019), these contexts usually present controlled environments in which the well being and development of the person with DDs is usually prioritized over organizational constraints. At the same time, there is limited work studying the introduction of robots in workplaces employing people with DDs, and this work is focused on specific aspects of the collaboration during a task. In our case, we see the practical need to go beyond this and understand the organizational properties as well as the perspective of every stakeholder in the company to recognize the potential impact of the introduction of our robots, to then guide our design decisions in a complex setting with vulnerable users.

### Value and Human Robot Interaction

The perceptions and values that designers or roboticists have about technology affect their view of “human,” “machine” and “robots” (Suchman, 2007; Wallach and Allen, 2009; Suchman, 2011; Richardson, 2015). Even though users experience robots and attribute qualities to them, designers and developers aim to include specific kinds of experience or quality in their design. This inherent bias—relying on their own preference—has been highlighted in research (Oudshoorn et al., 2004).

Design activities (Brandt and Messeter, 2004; Belman et al., 2011; Friedman and Hendry, 2012) and interviews have been often used as a tool to elicit values from the users with few exceptions (Fleischmann and Wallace, 2009; Fleischmann and Wallace, 2010), which were conducted with developers. Design fiction (Bleecker, 2009; Tanenbaum et al., 2012; Blythe, 2014) has been used in HCI as a speculative space (Blythe et al., 2016) that allows researchers to understand the societal impact of future technology (Blythe, 2017) and the values related to it (Dourish and Bell, 2014; Schulte, 2016; Muller and Liao, 2017; Wong et al., 2017). According to Fitzpatrick (2015), for designers to become “responsible,” they need to be reflective practitioners, aware of their power in inscribing futures. This is possible when designers’ and roboticists’ viewpoints are also considered and made explicit before design. HRI has mostly used narratives, scenarios (Sung et al., 2009) or stories for feedback from intended users on design concepts and prototypes (Robinette et al., 2016; Lichtenthäler et al., 2013). Surveys (Fong et al., 2003), scenario-focused workshops (Caleb-Solly et al., 2014) and sketching of future scenarios (Sung et al., 2009) have been used to study the perception and needs of the users and to evaluate robots.

Robotics researchers involved in previous studies have documented their opinions as robot experts (Scherer, 2014), when evaluating robot prototypes (Sauppé and Mutlu, 2014) or by participating in design sessions (Lee et al., 2014). Cheon and Su (2016)’s study shows that roboticists’ engineering background influences their views on the design of robots. Futuristic stories (Cheon and Su, 2017) and futuristic autobiographies (FABs) (Cheon and Su, 2018) have been used to understand the values of roboticists. Futuristic autobiographies, inspired by design fiction, help to elicit values and perspectives from participants such as prospective users, designers, and researchers (Cheon and Su, 2018). They have been proven to indirectly help us understand the values by examining how participants see the proposed situation and map out possible actions. In our research, we used design stories inspired by FABs and this contributes to the existing body of work using design fiction as a research method in HRI. Through our work, we want to understand how stakeholders perceive the future with respect to people with DDs using robots. Though design that reflects on values and ethics has been stressed (Holmquist and Forlizzi, 2014), there is a lack of guidelines for ethical or responsible robot design (Cheon and Su, 2016). We want to go beyond solving the problem of designing current technologies to explore the social and ethical implications of these technologies, incorporating the views of all stakeholders.

## Understanding the Setting

The foundations of design are often best built on a clear understanding of the people, settings, and purposes you are designing for—this reduces mistaken understandings and beliefs and often provides better insight and orients you to the needs of the people you are designing for. In particular we wanted to qualitatively evaluate the social, organizational and technical operation of our partner organization, and consider what sort of problems design could address, how people doing particular activities with particular needs might be supported, or how an innovative concept might either mesh with or disrupt particular work or activities.

In the first phase of our project, two researchers from our team undertook an ethnographic study consisting of three days of observation of the activities of the company (which was engaged in managing a coffee shop, a printshop, a flower shop, a bakery, and the local delivery of in-house products) and semi-structured interviews with the CEO, the educational team (equivalent to HR) and managers from each area.

Ethnography (Martin and Sommerville, 2004) is specifically designed to provide a rich understanding of social phenomena as they occur in everyday settings (Randall et al., 2007). It is qualitative in nature and involves interviews, observation, and participation in natural settings with the specific groups of people you want to study. Our orientation to ethnography is ethnomethodological (Garfinkel, 1967; Garfinkel, 2002) which means that it is not theory-driven but rather focuses on revealing and describing the way in which the people we study organize their activities and their understandings, closely related to how they ordinarily do things themselves, and aiming to minimize the use of technical language. This means that the work stays close to the lived reality of the natural phenomenon itself and that the products of the research are easy to understand across disciplines, which make this approach particularly useful in multi-disciplinary research. While three days is a short period of time to do a full study of all the activities, it was in this case sufficient to provide a good sense of the overall organizational structure and the relationship between its parts. This approach is close to what (Millen, 2000) describes as “rapid ethnography”, which sacrifices depth of understanding for a more focused assessment targeted at key individuals and functions.

Ethnographic data can take different forms: general descriptions of behaviours, descriptions of physical layouts, close descriptions of conversation, thoughts and feelings about what is going on, tentative hypotheses, examples, repeated occurrences, responses to questions, etc. While it can be possible to generalize learning beyond the specific context we are looking at, a more essential analytic choice when engaged in a collaborative design activity is to reach a representation of the activity (and all its elements, including technology) at the heart of our specific scenario that is shared by and recognizable to all the stakeholders.

To understand the organizational and socio-technical properties of the setting or scenario we are looking at, we infer motives, purposes and rules of conduct, and give meaning to the activities we observe. We take these elements to be normative, not causal. They do not exist independently of context and are bound up with the cultures, traditions, plans, etc. of the setting we are dealing with. So analytically what we attempt to do is explain them such as they are adopted, observed, recognized and understood, enforced, broken, etc. by the people in that setting.

Through our ethnographic study and semi-structured interviews, we understood that the self-described goal of the organization we were collaborating with is to show the value of disabled workers and demonstrate that it is possible to provide them with gainful employment, given the appropriate organization of the workplace. Indeed, the CEO told us that if other companies were to learn from them how to manage employees with DDs, her organization would no longer need to exist. They have over two hundred employees with varying degrees of DDs in different business units and run most of their operations at a profit. They go to great lengths to deliver products which are indistinguishable from what might be provided by any other print-shop, florist, or bakery, and with a very short turnaround period. They achieve this by breaking down their workflows into basic tasks and implementing a strict division of labor. This means that many of their employees are engaged in repetitive activities requiring limited initiative or creativity, basic coordination of tasks (as found on a production line), and little need to deal with unexpected occurrences. Tasks requiring more complex social interactions or creative choices and responsibilities are, with few exceptions, handled or closely supervised by non-disabled managers.

An example of this can be found in the flower shop business line developed by our partner organization. This unit is not a brick-and-mortar flower shop with a customer facing physical location but takes business-to-business orders (via phone and e-mail) for flower arrangements and fruit baskets. As mentioned above, the shop operates somewhat like a factory line, where the activities the employees with DDs undertake are broken down into basic tasks. More technically complex tasks such as back-end ordering and invoicing are handled by non-disabled employees. Interestingly from our point of view, more creative but not technically complex tasks requiring, for example, aesthetic judgment, were also mainly handled by the manager. This left most employees performing mundane tasks such as folding ribbons.

A similar scenario can be found in the printing business unit. Like the flower shop, this unit takes mainly business-to-business orders for a variety of print jobs, with a large proportion of these being for business cards. With the print shop having what appears to be a larger variety of jobs, we also observed a broader variety of tasks involving specialized machinery, such as cutting and binding. But the fundamental principle of breaking jobs down into basic tasks and implementing a division of labor remains. As each step of the process is relatively simple on its own, the likelihood that mistakes would be made that might compromise the quality of the final product is minimized. Furthermore, this kind of division of labor creates a collaborative and social environment without creating a need for complex and potentially stressful communication and coordination of dependent activities.

We did observe some exceptions to the way work is organized (as described above). The print shop had one person who managed the print server for one of their digital production presses. This is technically complex work, and the manager of the service explained to us that the technical literacy of the individual combined with his curiosity led him to that role, but that they wouldn’t otherwise try to encourage employees to take on more complex tasks. However, pre-defined employment criteria mandate that all employees have the skills to independently navigate to and from work and be able to use standard technology such as phones, TV, etc. In the flower shop, some employees are encouraged to fulfill orders for certain types of potted plants on their own. This is certainly more complex than folding a ribbon, as it involves several steps, and a degree of aesthetic judgment to decide that the final result is good enough. But bear in mind that even the aesthetic judgment involved here is “reduced” to repeatable instructions, such as measuring the distance between different parts of the composition to ensure consistency and balance or symmetry.

The question as to whether more or even most of the organization’s employees would be able to learn to adequately perform more complex and creative tasks, given time and attention, was not, we were told, seriously considered by the organization’s managers. This was not due to indifference toward the employees’ personal development, or (as the examples above demonstrate) a lack of ability to recognize talent where it exists. What was clear was that the viability of their commercial operations had to take precedence over individual learning and development. Consequently, the assessment that employees with DDs cannot handle uncertainty and exceptions is not a clinical judgment or even a character assessment but is appropriate to the requirements of an efficient workflow.

Further evidence of this can be seen in the handling of the one operation that was openly handled at a financial loss and was therefore not subject to the same operational constraints: delivery. The organization has employees personally deliver some of its products (like business cards) to its customers in the metropolitan area. Employees currently assigned to the delivery service are given at the beginning of their shift a backpack, the name and address of the recipient, a receipt form to be signed by the recipient, and a cellular tracking/communication device that they can use to call for help should the need arise. They are then essentially left to their own devices to find their way to the delivery address and back.

We were given the opportunity to accompany one of the employees on a delivery run and were able to make some, to us, revealing observations. Most notably, the employee in question had developed a very nuanced understanding of the vagaries of the underground transport system, and was able to determine, for example, which carriage would allow him to descend closer to the escalator at a connecting station, and to memorize the complex layout of different stations across the network. This demonstrated initiative and a degree of creativity or inventiveness on his part. This was not a new insight for the managers of the service. After all, being able to make their way independently to and from work is required to be hired by the organization. The service also offers employees an opportunity to interact with people independently and with purpose and seems to foster a sense of pride and accomplishment. In spite of it being a mostly individual task, the delivery service also creates opportunities for socialization, as the employees often leave the office together and may travel together for a distance, and even help each other in the case of new or less confident employees.

This is to say that we do not intend to overstate the somewhat Taylorist character of the work of our partner organization, and that we are not implying judgment of their ethics and their overall mission. Their agenda is to demonstrate that gainful employment and financial independence are possible for employees with developmental disabilities, and on their own terms they appear to be quite successful. We can also observe, although this was not central to our project or our analysis, that the Korean work culture (and perhaps Korean society at large) is quite hierarchical and can ask individuals to subordinate their role in the workplace to shared goals and outcomes. From that point of view, what is experienced by the disabled employees we observed could be considered relevant training for what they could expect to experience in other workplaces, i.e., to contribute to the organization rather than lay stress on individual development.

We are nevertheless aware that the operational concerns we witnessed could compete with the clinical and educational configurations of the worker, and that more flexible assessments of the employee’s ability to handle uncertainty and develop skills might conflict with concerns about disrupting existing workflows. As researchers, we bring concerns and biases of our own to a future scenario. The most obvious is that being part of an organization that prototypes modular robotic platforms, there is a technology push toward making our platform fit the scenario. This for us is not just a matter of persuading stakeholders that our technology is good or desirable. The introduction of AI and automation to a workplace (and a robotic platform potentially embodies both) is not an ethically neutral action, and how you design the technology is inevitably driven by a vision of what you believe the role of the people and of the technology involved should be.

As HCI researchers, we were particularly struck by the limitations in terms of creativity and the potential for personal development that the organization of work we witnessed can engender. We wondered therefore whether there was an opportunity for robots to enhance the work experience of employees while maintaining the efficiency of the service. Robots could assist the employees to perform their tasks more efficiently but always respecting their role in the process, prioritizing their social and professional skills above process optimization. Robots with an appropriately designed information management system or interface could also enable new types of tasks by providing a structure that standardizes activities not currently performed by the employees due to their flexible nature or a higher level of complexity.

The subsequent step, which is detailed in the next section, was to try and bring as many of the stakeholders as possible together and try to tease out ethical considerations and perspectives around what role and responsibilities robots might have in this workplace, with the aim of converging and agreeing on a potential, concrete scenario.

## Design Approach

Our proposed robotic platform can independently navigate complex, crowded environments and could be used to transport and deliver objects. The challenge for us was to identify the need for robot collaboration within the service and to propose effective solutions by adapting the functionalities of the robotic platform.

In prior research on robots being introduced among people in workplaces (Mutlu and Forlizzi, 2008; Dietsch, 2010; Smids et al., 2019), it was found that robots may affect social settings and be interpreted as displaying social behavior simply by being there and acting among people. The envisioned future of robots working alongside DD employees requires careful consideration of the organizational, ethical, and societal consequences and values related to robots.

We conducted participatory design sessions with two managers from the company we studied, four engineers in charge of developing the robots, and four designers whose task it was to define and shape the interactions between humans and robots. During the session, we introduced the capabilities of our robotic platform (it has a touchscreen for interaction and is able to navigate autonomously, detect obstacles, carry items) and the participants assessed our technology’s feasibility in various services. They selected the service for which the introduction of the robotic platform would be considered most beneficial. The participants outlined the service’s challenges and proposed concepts to solve the challenges thus identified. These proposed concepts were assessed relative to the capabilities and limitations of our robot.

We acknowledge that just eliciting design requirements is not enough for a use case that is ethically complex. For the design of our service, it was important to understand the perceptions and values that designers or roboticists have about technology, which affect their view of “human,” “machine” and “robots” (Richardson, 2015). The technology stakeholders have different values, which they feel very strongly (Knobel and Bowker, 2011). They aim to create a specific kind of experience or quality in their design. Practically speaking we are facing the challenge of finding a robotic deployment scenario which balances the technological ambition of our own organization with the business model of the recipient one. The disabled employees themselves are potentially caught in the middle of these ambitions.

Bell and Olick (1989) stated that society is re-created each day as people act, calling on both their memories and anticipation. “Arguably, our job as the futurists designing the narratives, is to make the process of re-creation or re-imagining of the society more conscious.” During the workshop, we also conducted a value elicitation exercise with the participants. We used futuristic stories, inspired by futuristic autobiographies (FABs) that allow us to understand the societal impact of future technology and help elicit values and perspectives from participants such as prospective users, designers and researchers (Cheon and Su, 2018). By using this method we aimed to restore the future users, people with DDs, to a central position in the minds of our participants when anticipating, designing, and evaluating the future of robotics.

We wanted the participants to go beyond passive imagination and own the futuristic fictions we created. Futuristic autobiographies have been shown to be an effective means of eliciting rich narratives that incorporate participants’ experiences, practices, and viewpoints. While design fiction has been defined as the deliberate use of diegetic prototypes to suspend belief about change (Bosch, 2012), in FABs the participant becomes “diegetic” (Cheon and Su, 2018). Instead of having the focus on or around a prototype, the focus of FABs is on the participant itself. We preferred the autobiographical style where these fictions are not perceived as “too abstract” and could be given new meanings through each individual’s experience. Unlike previous research, which uses this method only on roboticists, the FABs we created were crafted with the intention of using them on different stakeholders (executives, designers and roboticists).

We conducted the FABs with eight Korean participants, six male and two females, between the age of 24 and 50. Two (P1, P2) participants were managers from the Korean organization employing the people with DDs, who had no prior experience with robots. The other participants were from the robotics organization. Two (P3, P4) were User Experience Designers responsible for ergonomics of the robot and its interaction with people and the remaining four were robotic engineers (P5–P8). Our participants were selected to represent the stakeholder groups involved in our project.

Each participant was presented with three FABs that were specifically designed according to their stakeholder group. We used the guidelines and cautions presented in Cheon and Su (2018) to create our FABs. The authors researched each participant’s background (prior observation of their tasks, their portfolio of work and research interests) to create the FABs for the stakeholder group. These were less than 80 words long, with interesting and plausible scenarios which facilitated open-ended discussions on multiple themes around work collaboration of robots with developmentally disabled people. Some FABs overlapped between the stakeholder groups as they had aspects of information pertaining to both groups. It was also interesting to analyze differing viewpoints about the same scenario. An example of the FAB presented to managers and designers is: “*Recent declarations have caused concern among the executives of the organization. Many employees have stated that they prefer to collaborate with robots as managers rather than other human managers. Who operates the robots? Is it the managers? If yes, what kind of control was given to the managers to determine robot actions? Why would employees prefer robots over humans?*.”

The FABs were conducted by four facilitators (authors of the paper) on the premises of our organization. Seven FABs interviews were conducted in English and one was conducted in Korean and concurrently translated by a facilitator to English. The interviews were held in small meeting rooms and were audio recorded. Each session had one participant and one facilitator and lasted 20–30 min.

The interview data was open coded in turns by two researchers to generate themes. For example, they were coded into categories of “perception about robot,” “giving human attributes,” “role of technology,” “safety,” “privacy,” etc. These themes were reiterated through discussions with other researchers. Our analysis focused on how the participants responded to ethical and social questions regarding the role of robots, its users and their behavior.

## Findings

Participants responded to futuristic stories where workplace collaboration between robots and people with DDs is an everyday task. They imagined the type of robots, their intentions for building or deploying them, the tasks performed by them and their impact. The findings cover the emerging themes of human-robot collaboration and the potential positive and negative consequences of introducing robots.

### Roles of the Robot

#### The Robot as an Assistant

Participants imagined very specific tasks: “robots that carry heavy stuff, guidance robots, surveil-lance robots” (P8 - Engineer), “cleaning robot” (P7 - Engineer), “delivery robot” (P6 - Engineer), to more generic tasks like helping in everyday activities in the workplace. Robots will help in enhancing the capabilities of employees and supporting them in doing more. They discussed examples of robots helping them in their current tasks and undertaking new tasks, such as sharing meeting notes, etc. (a job only done by the management).

“*The robot will help people in their capabilities… to increase their capabilities. For example: processing information and maybe provide navigation*” (P6 - Engineer)

“*They can also arrange another meeting and share meeting minutes with other people. Like just ordering the robot like please, send some meeting minutes to someone*” (P7 - Engineer)

#### The Robot as a Collaborator

Participants also saw the robots as a potential team member who complements the job of the employees with DDs. It supports their job like a partner, being more collaborative and going beyond just enhancing abilities. Participants imagined a positive relationship with collaborators as they will help the employees to do more. The robots and the employees will complement each other and overcome their weaknesses, such as picking up a screw for the robot and forgetting the correct placement for materials for the employees.

“*For example, there is a robot that screws small things ten times. Maybe the robot can turn the screw exactly ten times, but grabbing the screw is not possible with the current technology. So maybe the employees can help with those things so that final job is done by the robot, but it is sequential, and they complement each other with their different abilities.*” (P2 - Manager)

“*This would ultimately lead to the employees becoming confident. They get feedback from anywhere, including from robots, managers, and other sources. So, there are more things that the employees could do voluntarily.*” (P1 - Manager)

#### Robot as a Supervisor

Some participants believed that robots will be successful only if they are more intelligent or if they behaved more intelligently than the people with DDs. They imagined the robots to be like a manager, either replacing or helping them in their existing tasks such as counseling and logging. In these situations, the robots were perceived to be superior because replacing or collaborating with the managers placed robots on a similar work-hierarchical scale of the managers. While robots were perceived as positive for the employees as an assistant and a collaborator, some negative consequences of the robot working as a superior were imagined in the supervisory role.

“*In the (….) meeting they (people with DDs) can talk, and the robot can write down everything they are saying. Then the counselor (educational team member) can analyze the meeting minutes or the logs.*” (P7 - Engineer)

“*They could be surveillance robots. And as surveillance robots, they could be seen as enemies by the employees because they will be watching them and will report to the managers what they did wrong and stuff.*” (P8 - Engineer)

### The Human-Robot Relationship

The conversation about the roles of the robots was often complemented by how participants perceived the robot. It went beyond a technological artifact to referring to it as an individual. Participants described robots having an identity higher than a tool. They highlighted a need for establishing good and bad behavior of humans with the robots.

“*I want them to be equal. And sometimes we have to think about robot rights like human rights. I want the robots to evolve to that level of (humans in) cognitive power and physical abilities.*” (P5 - Engineer)

“*So, in this case (employees hitting robots) it’s not about robot or human, it is about someone who cannot hit back. I think it’s a problem of human behavior so we have to control their interaction, the people and not the robot.*” (P2 - Manager)

Participants discussed the robot’s likeness to humans in terms of physical appearance, cognitive skills and actions. This is in line with the theory of regulatory fit [13] which states that an agent (in this case, a robot) that adapts to people’s orientation might elicit more cooperation than someone who doesn’t. For successful future collaboration, the robot has to have a developed cognitive power capable of “understanding human intentions and emotions” (P7 - Engineer), be “self-learning and updating” (P2 - Manager) and give “more human-like feedbacks” (P1 - Manager).

“*If it is following the human employees, I think that it’s a kind of pet or it could be a machine or a human; even though it’s a machine, they react as if it’s an live object. Then they could act toward the product like a semi-human being. They try to speak.*” (P4 - Designer)

“*I want the robot to behave like a person. When you pass by other people, they communicate with gestures, faces. I want to imagine that the robot communicates with the people the same way as humans do.*” (P6 - Engineer)

While several participants voiced a concern about robots replacing the job of humans, others did not see robots as a threat. They believe robots will prove to be useful and hence can be seen as friends. Others stated that the robots will bring anxiety and fear in humans because of their power. This might create a larger divide between robotic experts and other people, including people with DDs.

“*Robots are smart enough to recognize that if people are not following their instruction, then they can give them, not a star but a negative of a star and you can keep track of it.*” (P8 - Engineer)

This was said in the context of how to make people respect the authority of robots, by building rewards and performance management strategies into the human-robot relationship.

### Autonomy and Control

Participants stated that the efficiency of robots is equivalent to its autonomy in navigation, decision making and achieving self-diagnosis. Robots were deemed useful when they bring automation into the process.

“*At first we should make the robot survive in this world without any special help (from the developers). Engineers have to be there always so it cannot survive by itself. You have to charge it. When it goes the wrong way or stops somewhere, put them on track manually. I have not thought about it beyond that, so I really focus on making the robot self-smart. And I actually don’t think much about what it can do for us.*” (P5 - Engineer)

“*I am assuming that robots will be autonomous because if there were a manager to each robot then that would be way too inefficient unless some part of it is automated.*” (P3 - Designer)

In the context of the workplace collaboration, the robot was unanimously thought to be controlled by the managers of the organization, although participants had differing viewpoints on the type of interaction and the level of control given to the manager. Managers were understood to have control over the robot’s autonomy in different tasks.

“*I am assuming that the kind of control given to the managers would be to designate roles, criteria to focus on, and maybe limit its functions with respect to people’s privacy or limiting its function to respect the roles that the humans have.*” (P3 - Designer)

“*Because we (robot experts) cannot control remotely the robot. But this robot should have some kind of intelligent things like autonomously moving or AI chatbot. So, anything can happen. So, someone has to control and maintain the robot. I think that person should be the manager.*” (P4 - Designer)

“*Managers will operate the robot. Who else would?*” (P8 - Engineer)

A few participants also spoke about the safety of deploying robots. They defined this as dependent on the task carried out by the robot. The nature of the task would define appropriate “safety levels” that needs to be thought before implementation. Participants also reflected on privacy of any collected data by the robots. They were unclear on what is ethical in terms of data privacy, an aspect that they admitted to not have considered before. The robot’s understanding of “sensitive” and “appropriate” information was also questioned through the FAB narratives.

“*Maybe it can harm people. It cannot counter its force. So, for example when it can give a high-five to people, someone’s arm could be broken. It can also go and crash something*” (P7 - Engineer)

“*I am sure it’s going to be more of being watched more precisely. Because in the past, the bosses, the big brothers were always watching people. But they were only humans. But robots can be everywhere. And they don’t get tired. So, it’s going to be more threatening.*” (P8 - Engineer)

“*They just write down all that is said during the meeting, but some part should be erased which is due to security reasons or some small talk. The robot doesn’t need to write it or talking bad things behind someone. Robot can’t share it all.*” (P7 - Engineer)

Values are context and people dependent. As in this case, privacy is a value held by a person (people with DDs) or it can be held by the organization. It can be intentionally embedded within a technology (monitoring) or materialized by the context of human interaction (writing in meetings).

### Value in Terms of Business Needs

The business motivation and needs of this project were rarely forgotten by the participants. On the contrary, business costs, success and issues of profitability were brought up by all participants in one or more discussions. In cases where the futuristic decisions went against the business needs, participants expressed genuine concern. The justification of deploying robots even in less-than-ideal futuristic stories, was often based on a decision about profitability.

“*The employees and the organization have great expectations about collaborating with the robot because it can reduce the workload or somehow have good effects. Somehow the reasons for shutting down (the robot collaboration) is different from the needs of employees or the organization.*” (P2 - Manager)

“*And I don’t know ultimately if not having managers will be profitable for the company and there will be more money to be shared among the employees. So, I guess reducing labor will have cost benefits.*” (P3 - Designer)

“*Yeah because using the robot must be cheaper or more efficient than hiring more managers. So, if problems happen, you will have to send for me anyway and that will cost a lot*” (P5 - Engineer)

While participants discussed robots replacing jobs, it was always the manager’s job that was thought to be replaceable, not the employees with DDs. This is also aligned with the organizations mission statement to provide more employment to people with DDs.

### The Eventuality of Robots

All the participants agreed that robots would be an everyday phenomenon in the future. The eventuality of robots coming into existence was compared with the likes of the industrial revolution and the “digital revolution.” This is in line with the technologically deterministic framework (MacKenzie and Wajcman, 1999) of dynamics between technology and society, where society fills a passive role of accepting and adapting to the results of technological innovation. They seemed confident in the ability of users to “adapt” to the new technologies (with a few exceptions).

“*But as you already know, in the early 20th century, we already faced the industrial revolution. So, every businessman would like to reduce the costs of cleaning and other things. So if they just, buy one robot, then can replace 10–20 human beings. Then they can reduce the cost dramatically. This means that people cannot stop developing robots.*” (P7 - Engineer)

“*Maybe 10 or 20 years later, because nowadays people live with smartphones right, the kids. After 20 years, I think the customers can learn and adapt. It takes time, it just takes time.*” (P2 - Manager)

“*Some other generation like the elderly people like they can be afraid of this kind of new technologies. They can’t imagine living with the robots. So I want them to use the technology easily. But somehow, someone is not able to. There can be a generation gap.*” (P4 - Designer)

In this eventuality, participants discussed the changing behavior of humans due to the collaboration with robots. They were asked to imagine any behavior that could possibly be “unnatural”. They defined what is “unnatural” for them and how it would affect people with DDs through their narratives.

“*It is unnatural that the human follows the machine*” (P8 - Engineer)

“*If they started serving more human tasks that require more emotional attachment but is still in the form of a tool kind of, even if that’s the case, people could feel like it’s unnatural.*” (P3 - Designer)

One participant imagined people becoming extremely dependent on robots, causing them to lose their survival skills. This prediction of the future suggests the removal of the societal norm to work. This means that people with DDs won’t need jobs as a measure to assert independence or build their skills.

“*I think many people will think that robots will make people lazy. For example: if robots cook for you all the time, all the human chefs will lose their jobs and after 100 years no human will be able to cook their own meals. If something happens to the robotic systems in the world, many people will die from hunger because they cannot cook. People might worry about this. Robots might remove the human ability to survive on their own because they will rely on robots for everything. More than society, everyone believes that you have to work to get paid and robots might change that so even if you don’t work, robots can do all the jobs for you and you can play all the time.*” (P5 - Engineer)

Hence, participants in this context defined “unnaturalness” as a disruption of normal social relations and hierarchies which would have to be carefully handled when incorporating robots in the future. Unnaturalness as discussed with participants can have extreme consequences. People with DDs struggle with “normal” or “natural” routines. In case of robots doing all the works in the society, people with DDs would not need to struggle to learn “old survival” skills but will have to invest themselves in learning the new “survival skills” of operating a robot. This can also lead to extreme alienation of this vulnerable population.

## Design Considerations

The two-phase study of ethnographic observations and the design workshop with the FABs activity enabled a deep understanding of the organization, the users, and the ethical perspectives of stakeholders. In this section, we outline some of the design considerations we defined from this understanding.

### Robot Roles in the Organization

During the FABs interviews, the executives and designers favored robots that were helpful in the tasks performed by or for users. Also, all participants justified the use of technology for being cost-effective and catering to business profitability. The executives, being true representatives of the people with DDs, stated that they would discontinue using robots if they become a physical or emotional threat to the employees. However, other participants believed that any loss of humanness by using robots is a small sacrifice for higher capabilities, productivity and power. Indeed, when looking at the company, most services adhere to a serialized organization of their processes that results in simple and repetitive tasks for each employee. This serialization reduces the occurrence of unexpected situations guaranteeing the emotional safety of the employees and also maintains the consistency of the outputs.

In this organizational structure, we identified a tension between the opportunities for personal development of the employees and the viability of the commercial operations. Robots, as assistants rather than supervisors, could effectively mediate between these two objectives, enhancing the work experience of employees and the possibilities for social and professional development while maintaining the efficiency of the service. At the same time, robots could enable new, more challenging or creative tasks, by providing a structured framework for activities that are not currently performed by the employees due to their higher level of complexity.

### User Characteristics

The intended users, imagined by the participants during the activity, were both the managers and people with DDs. Managers were seen as primary users, while people with DDs were seen as secondary users of technology. This viewpoint of robots’ design and use by the managers raises the issue of a socio-technical gap. In a case study (Grudin, 1988) of organizational interfaces or “group-ware” applications, the challenge of uneven distribution of benefits of these applications among members of the organization stands out. While these applications are designed to provide a collective benefit, some members of the organization may need to do more work, which may result in the rejection of the system. If we consider robots as a version of a new age “organizational interface,” these uneven distributions might be more pronounced. While the managers are also new users to robotic technology, people with DDs might be affected more as they will go through higher adjustments. It raises the question, “Are we marginalizing the people with DDs?” Hence, we identified the need for simplistic and relatable interfaces and structures for both managers and employees to perform tasks.

Another aspect highlighted by the executives during the study was the passiveness of being a technology consumer. When discussing the problems a robot could have, P2 said: “But I don’t answer any more because it is not a problem from our side.” This reveals the dependency of users on designers and developers of technology and the vast responsibility they undertake without being aware of it. Technology development is often left in the hands of experts (Šabanovic, 2010) and hence understanding and mediating the values of these “experts” with those of the users using human-centered approaches become very important.

### Adaptive Autonomy

Participants saw the robot as a collaborator that would enhance the work of the employees. They should perform tasks autonomously, but also side-by-side with the employees to overcome each other weaknesses. Participants mentioned that, in some cases, a human should take control of the robot leveraging her/his existing knowledge. *Sliding Autonomy* is a strategy that integrates the autonomous capabilities of robots with the reliability that human control can bring in the completion of complex tasks (Heger and Singh, 2006). By changing the level of autonomy of the robot, this strategy makes it possible to leverage and combine the capabilities of humans and robots and adapt the degree of control to address new or unexpected situations (Tang et al., 2016). In the case of our robotic platform, this is an aspect that could be considered to address technical limitations. For instance, navigation could be dynamically adapted, allowing managers or employees to temporarily guide the robots in specific situations, e.g. to overcome obstacles or find the way through a crowded space.

Most participants imagined the person controlling the robots to be the manager. They had, however not imagined negative consequences of this control, like “increased sense of surveillance,” “abnormal workplace hierarchy,” etc. While control should be given to the managers for a few complex tasks or in specific instances respecting the workplace hierarchy, an environment of distrust between the employees and the managers should be avoided. Hence, the control of the system should be distributed among all its users rather than a subset.

Beyond this, adaptive support could be provided by the robots to generate new learning opportunities for the employees with DDs. By gradually decreasing the level of support the robot provides during the tasks, employees can increasingly gain independence in their work. However, the option to rely on a higher level of support remains available.

### Managing Expectations

Through our research, we were able to identify the expectations stakeholders had of the robotic platform. While we found common points across groups (i.e., organization, managers, designers, engineers), it was interesting to identify the differences in their perspectives. As designers, we should be able to reach compromises that address these, sometimes, contradictory expectations.

In this case, all participants looked at future robots as a real work “partner.” Roboticists displayed a high level of attachment to robots and were passionate advocates of technology. Their take on the futuristic stories had technology as a central theme, and their utmost concern was indeed the level of technological advancement, which would enhance or limit the robot’s utility. Their imagined future problems were limited to “software,” “sensors,” “algorithms,” or “control system.” Executives were rather focused on the employees with DDs. They were conscious of creating and setting strict boundaries of user interaction with the robots. For example, the need to teach the people with DDs that hitting a robot is bad as these could translate to their real-world interaction with other humans. Indeed, collaboration with robots came with expectations based on the dynamics of human-human work collaboration.

All participants declared that if we wanted the robots to work with us, then we have to make them like us. This meant higher cognitive powers, ability to understand, process, respond to human actions and emotions humanely. However, the participants also appreciated and wanted robots to retain their “robotic” quality. “Robotic” quality was equated with consistency. Previous research has shown that when users perceive the robot’s actions to be less predictable, they anthropomorphize the robots more to reduce the feeling of uncertainty (Waytz et al., 2010). This brings out the different yet coexisting perspectives of wanting the robots to be simple and objective and yet more human-like. Designers were aware of the probability of users over-estimating robot’s technical capabilities as a major source of future discontent in the work environment. Indeed, managing user expectations is especially imperative in the current context of working with the vulnerable group of people with DDs. They are equivalent to the naive users discussed in Cheon and Su (2017), who have high expectations beyond what the roboticist intended to program. Often technology is introduced as a marketing gimmick that attracts and disillusions the users. As developers and designers of technology, it is important to manage user expectations through the physicality of robots’ form, shape, and size.

## Concept Proposal

Guided by the ethical considerations discussed in our findings, we defined the role of the robot to be the assistant of the employee with DDs. Indeed, it should not replace them in their activities, but rather improve the conditions of their work and augment human capabilities, aiming to increase their independence, agency, and learning opportunities. This was a central aspect for us and was in line with the concerns and expectations mentioned by the some of the participants during the FABs activity. Rather than replacing their dependency on managers with a dependency on robots (with a supervisory role), the robots should act as a support system ready to act in case of need. Also, as stated by the CEO of the company, the goal of the organization was that of employing the maximum possible number of people with DDs. This was considered a possibility by replacing or reducing the need of managers through introducing robotic automation. While, as designers of technology, we usually see the replacement of human labor as one of the potential risks of introducing automation technologies, in this case we found ourselves in a more complex setting with no simple answer, proving the importance of a deep understanding of every specific case and the involvement of every stakeholder in the process. Hence, a compromise was reached which was to reduce the ratio of managers per employee. It would reduce the costs and ultimately allow them to hire more people with DDs. These considerations were later considered when defining the features of the robot.

The plant management service was identified as the most suitable scenario for the introduction of the robotic platform during the participatory design workshop. The setting was ideal to incorporate automation to alleviate the workload of the managers while augmenting the skills of the employees by fostering independence and improving work conditions. In this service, teams of one manager and two employees with DDs manage the indoor plants of corporate offices around the city. They perform activities such as watering plants and cutting dead leaves with the manager having the additional responsibility of supervision. It is an activity that requires physical efforts, as they need to carry the water. Most employees work under the close supervision of their manager as even the most independent employees need regular advice for verification and unexpected situations (work emergency), which constitutes the following instances: spilled water, dead or fallen plants, and instances when questions were asked to them by the people working in the company they visit.

After identifying this scenario, we conducted additional interviews with the managers of the plant management service to complement the understanding we gained during the previous interviews, observations, and workshop activities. We performed a detailed analysis of the characteristics of the service environment, the tasks, the users, as well as the capabilities of our robotic platform, to identify the moments of intervention in which the robot could provide valuable support to the employees, and the best way to implement it.

The teams of one manager and two employees with DDs visit the plant management sites once a week and the time taken differs according to the number of plants it has (shortest: 40 plants >1.5 h and largest 350 plants >6–7 h). They need to perform their activities while employees of the other company are working in the office, so special consideration had to be taken to avoid disrupting their activities. The employees are trained through a booklet which includes the basic step-by-step guide to do each task (watering, cutting leaves, etc.). It outlines the plant’s need for water, sun, and shade with the plant’s picture and name. Sometimes employees carry this booklet for consultation. The managers give repetitive generic instructions about the plants at the start of each shift. The interviews and written instructions helped us construct a detailed work journey. The major challenges of the service for the employees and moment of intervention were identified through this activity: carrying the heavy bucket of water, filling the water, and controlling the amount of water for each plant, since it changes based on size and season; need for assistance on where to cut the plant if it is brown or dying, missing a plant that needs to be given water.

Our earlier conducted observation studies helped us understand the characteristics of the people with DDs employed by the company. Indeed, ‘people with cognitive disabilities can refer to a wide range of conditions that can go from very limited to high functioning individuals. We were able to understand the characteristics of the employees of this specific company by understanding their hiring process through interviews conducted with managers and HR. By requiring the ability to travel independently between home and work, and by asking to complete a number of manual tasks during the hiring process, they set clear boundaries for the capabilities that people with cognitive disabilities should have in order to work with them. The employees were transferred to different business units to acquire different skills. Additionally, the employees with DDs working in this service need to have certain specific qualities. There are often cases of outbursts (screaming, making loud noises, etc.) triggered by stressful situations given the conditions of the employees. A requirement for the employees of the plant management service is to a have very good control of their emotions, considering that they perform their tasks surrounded by the office workers of the client company.

We complemented our understanding with a questionnaire for the employees with DDs. The objective of this questionnaire was to provide a better understanding about the relationship employees have with technology and their perception about the robots. The questionnaire was filled out by ten employees (two females and eight males, aged 21–32 years old) who had worked in various business units. All respondents were comfortable using mobile phones, and most of them (8/10) used computers and TVs regularly. They used emojis (Figure 1A) to express their feeling and wrote down their impressions of how the robot looked. Most respondents were Surprised, Happy, or Neutral about the robots of our organization. They perceived it as a tool that could help them in general (move things around, guide people, give information, measure the environment), or in specific work activity (assistant in baking, automatic delivery, labeler for printing). Their positive responses indicated that they were curious about robots.

**FIGURE 1A:**
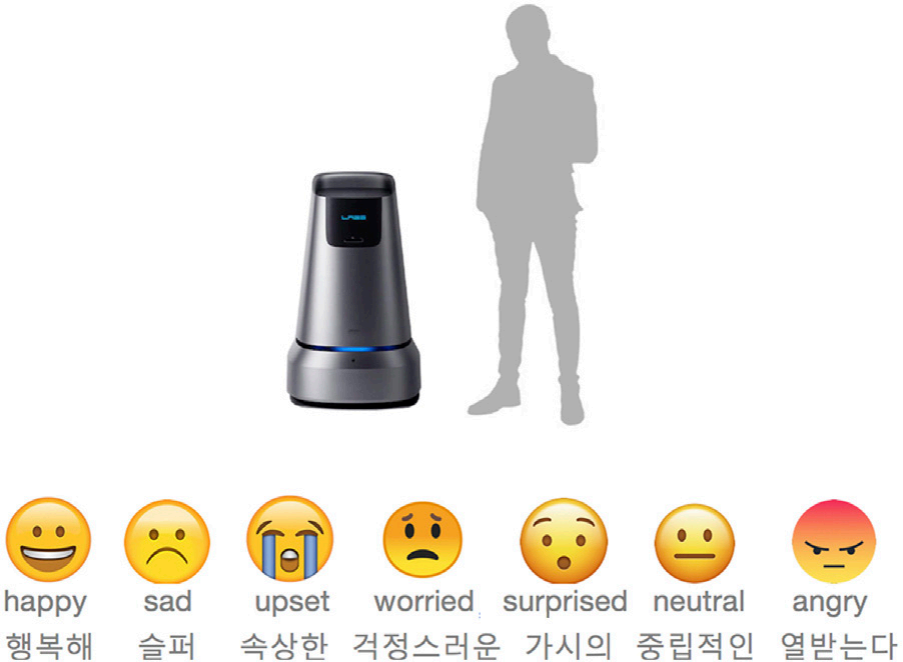
Snippet of the questionnaire filled by the employees regarding their perception about the robots.

Our robotic platform (Figure 1B) is able to carry objects, autonomously navigate through spaces and interact with the users through a GUI. We proposed a number of additional functionalities that we deemed required for this context. Indeed, the plant management service consists of several tasks that require different levels of automation and collaboration with the employee. The robot should be able to adapt to provide the right level of support to both managers and the employees. At the same time, we had to consider the special needs of the employees with DDs when defining the HRI elements.

**FIGURE 1B:**
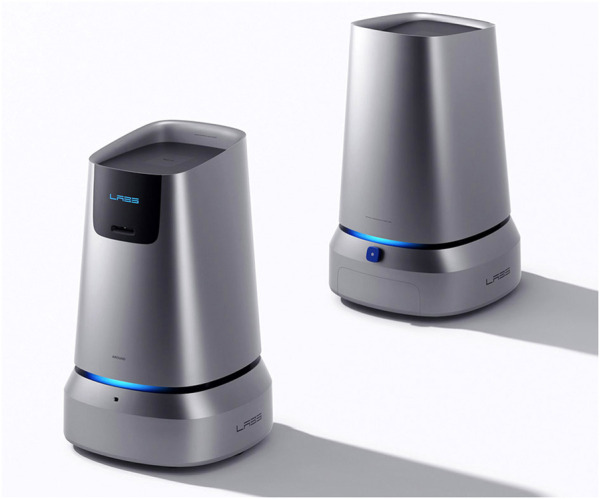
The robotic platform developed by our organization.

Based both on ethical considerations as well as on the specific characteristics of the plant management service, we proposed a robotic platform that would provide physical, cognitive and enable emotional support to the employees with DDs during their activities in the plant management service.

In terms of physical support, the robots would assist the employees by carrying the water, one of the most physically demanding tasks mentioned during the interviews by the managers. In order to do this, the robot would be motorized and able to move independently, as well as to follow the employees during their routes in the offices. Indeed, we proposed this navigation mode, i.e., the robot following rather than guiding the employee, to give agency to the employee over the robot and avoid putting them in a passive role.

The platform would also provide cognitive support by giving information about the amount of water needed by each type of plant, according to the humidity conditions and the season. Also, the robot would guide them on how to maintain the plants, especially which leaves to cut and how to do it properly. Aiming to facilitate learning, the information displayed would reduce progressively over time to allow employees to strengthen their own knowledge, with the robot ready to provide additional information or feedback when needed.

Finally, we planned the platform to enable emotional support for the employees, even in the absence of managers nearby. Employees would be able to easily request manager assistance in stressful situations through the platform. The managers would then be able to monitor and use the robot as a proxy to communicate with the employee and help them by assessing the situation and decide if additional assistance would be required. This type of remote monitoring and interaction would allow one manager to supervise several employees at the same time.

One important aspect to be considered in the following design stages, which was discussed during our findings, is what level of control would managers get over the robot and how they should handle the remote monitoring to avoid a sense of surveillance. It is possible to solve this problem by giving varying control of the robots to the manager. It means that employees and the managers are responsible for their individual robots and they can communicate with each other through the robots when required. To mitigate the sense of surveillance, the managers would oversee the location of each employee robot in real time when the robot performs only the autonomous tasks such as filling water and reporting to the office floor. Both the employees and the managers would have access to the shift report, which includes information such as the area covered (plants watered) in the office.

The physical aspect of the robots as well as the interactions are planned to present the robot as a tool and minimize any anthropomorphism to prevent any emotional attachments and false expectations regarding possible interactions. A touch and visual-based interface (buttons and screen) is proposed with screen and light feedback to maintain familiarity with the mode of operation by users.

## Conclusion

Throughout this paper we have tried to articulate some of the complexities involved in designing a robotic platform for employees with cognitive and developmental disabilities. We have taken a practical approach to resolving the ethical stakes in our project, and treated them, from a methodological point of view, as emergent issues rather than as *a priori* considerations.

We do not intend to suggest that high-level debates about ethical, responsible AI is not important. The themes that characterize current research on the ethics of AI, such as privacy concerns, responsibility and the delegation of decision making, transparency, and bias (Coeckelberg, 2020) are all very much pertinent to our scenario. But we also take on the analytic perspective that rules and norms of behavior are situational and negotiable, and therefore emerge and are made relevant in and through practice (Phillips, 1992). This is consistent with our tradition of ethnography-based design, and in our project, we therefore treated socio-technical and ethical issues as practical, emergent matters to be understood from the perspective of the actors we are designing for. And as Dewsbury et al. (2004) point out, an applied technology project moves forward not through political rhetoric but through recommendations for design.

As we pointed out in the introduction, the context-specific dimension of ethical questions requires attention that cannot simply be satisfied by high-level ethical principles alone. Everyone in our project can claim to have the well-being of the disabled employees at heart. And yet this does not mean that everyone will agree on what role technology and automation can or should play in ensuring that. Different parties will also be subject to different organizational imperatives or incentives. All this has to be unpacked and from a methodological point of view attention has to paid to the uniqueness of each project and scenario. This implies an analytic stance which is not necessarily oriented to the generalizability of the findings. And the people we involved in our study should not be though of as a “sample”—we do not examine our own motivation as researchers through a process of reductionism, and consequently we do not advocated doing so for other stakeholders in a collaborative project.

In terms of understanding the emergent ethics of our scenario, this project faced two broad challenges. The first challenge was that we had very limited access to the more vulnerable of our end-users, the disabled employees. While this is obviously not ideal, access to this population of users was owned and mediated by our partner organization, and this is also a scenario that is not uncommon in applied research conducted on behalf of and partnership with private and commercial entities. Given the nature of our partner organization, we knew they would most likely have their own mission statement with respect to their employees, and that our first responsibility was to understand how their own ethics influenced and were influenced by their organization of work, processes and managerial actions, which is to say how their ethics were practiced (Clegg et al., 2007).

The access we did have in the course of our brief observational study, along with the interviews with the service managers and CEO of the organization, allowed us to identify what, through the lens of our own agenda (to provide a positive role for our technology platform), appeared to be an interesting practical compromise between providing opportunities for personal development of the employees and the viability of a commercial operation. This for us represented an opportunity for proposing a technology design scenario that would enhance the intrinsic interest of the work itself for the employees without compromising (if not enhancing) the efficiency of the workflow.

The second major challenge, which is one faced by many technology design projects, is that the other stakeholders involved came from various disciplines (interaction and user experience designers, machine learning experts, and mechanical engineers) and were likely to have different agendas and ways of framing the ethical stakes involved. In fact, many of the people involved in our project (including ourselves) belong to professional categories that do not, for the most part, have a clearly defined professional code of ethics, and consequently may not have been in the habit of managing ethical considerations as part of their work in the first place.

The design exercises based on futuristic autobiographies, which we conducted with our stakeholders in the second phase of the project, forced everyone involved to confront each other’s priorities and concerns on the use of robots for people with DDs. It also allowed us to identify a broader set of ethical issues, not necessarily to provide a generalizable theoretical contribution to the ethics of AI, but to better manage the expectations of everyone involved while appreciating the risks of unintended consequences that were not obvious to us at the start of the project, and to ultimately agree on a shared scenario and set of features.

All of this is just a first step and careful and iterative empirical testing of the design concept will be required, ideally with more direct involvement of the disabled employees themselves, as we would not claim that preliminary studies and participatory design will ever allow us to anticipate all the ways a technology might be appropriated and how things might go wrong. But we do hope that we have made a compelling argument for the value of a practice-based understanding of ethics and that our discussion of how we approached the challenges in our project from a methodological point of view has been informative.

## Data Availability

The raw data supporting the conclusions of this article will be made available by the authors, without undue reservation.
