# Supplementary material for: Ethical Design of a Robot Platform for Disabled Employees: Some Practical Methodological Considerations
Source: Front Robot AI. 2021 Aug 19;8:643160. doi: 10.3389/frobt.2021.643160 (PMC8417308; doi:10.3389/frobt.2021.643160)
Supplement: Supplementary file 1 [file datasheet1.pdf]

Supplementary Material

1 Futuristic AutoBiographies (FABs) used:

1.1 FABs for Roboticists

1. Sometime in future….Today during a user experiment with your robot, there was an unexpected finding (malfunctioning). This will cause a delay in the deployment or possible discontinuation of the robot that you had planned to launch for people with impairments. What was this finding?

2. Recently, there have been mass protests about the robots that you have been developing. They are arguing that it is unnatural and would change the behavior of humans. Why are they protesting? In what way will this behavior change happen?

3. A few years from now, the robots you designed work alongside people in a workplace setting. It took people some time to accept them as your work colleagues. Now, they don't have a problem working with the robots. What kind of robots are they? What challenges did they initially face? What specific tasks are given to these robots? What process or work do they mainly contribute to? Do they collaborate with people with impairments? If yes? How?

1.2 FABs for Managers

1. It has been five years since the robots and people with impairments have been working together. Initially, you felt weird, but now you are comfortable with robots in the workplace. Why did you feel weird initially? What tasks do the robots do? How do they collaborate with the people with impairments? How did this impact your perception?

2. Recently, robots have been deployed in the workplace with people with impairments. These robots have been thoroughly tested and declared safe by you and the team for human collaboration, especially with people with impairments. But now you notice some behavioral changes in the employees. What are the behavioral changes that you have noticed? Did they all change their behavior? What were the factors that led you to approve the robots overseeing the cause of the problem?

3. Robots were introduced in convenience stores and worked collaboratively with people with impairments for a few years. Employees were satisfied with the robots. However, robots have recently been shut down and removed. The decision came after unending customer complaints. What were these complaints? How does the decision affect the employees and the organization?

1.3 FABs for Designers

1. Recent declarations have caused concern among the executives of Bear Better. Many employees have stated that they prefer to collaborate with robots as managers rather than other human managers. Who operates the robots? Is it the managers? If yes, what kind of control was given to the managers on robot actions? Why would employees prefer robots over humans?

2. After weeks of working with robots without problems, store managers have recently started reporting incidents. Employees with impairments have shown signs of stress after interacting with the robots, and a few of them have suffered outbursts, even reaching the point of hitting the robots. What caused these negative reactions? Why did things change suddenly after weeks of successful collaboration? How do employees interact with the robots?

3. Recently, there have been mass protests about the robots that you have been designing. They are arguing that it is unnatural and would change the behavior of humans. Why are they protesting? In what way, will this behavior change happen?
